# Letter from Will J. Kellett, of the N. Y. Press Association

**Published:** 1876-03

**Authors:** Will J. Kellett

**Affiliations:** "Wandering Willie," 84 Carleton Ave., Brooklyn.


### EDITORIAL. ETC.

N. Y. Press Association, Feb. 20th, 1876.

*Editor American Journal of Dental Science:*

Dear Sir—

In the December issue of the American Journal of Dental Science, among your editorials, you published a reply of an apparently much abused person—denying the facts stated by me in an article in the "*New York Evening Express*," of August 12th, 1875, entitled, "Dentists to the Front," and from which you transcribed and published in your Journal of the September issue. This person claims that the article was an anonymous communication. When to the contrary, the writer was the accredited Long Branch correspondent for the paper in question, as well as agent for a Press Association, writing over the *nom-de plume* of "Wandering Willie," a *nom* known to every literary person of the present day, and to which, as yet no shame or lack of fairness has ever been attached.

I simply published the article referred to as a "News Monger." I was not "seen" nor persuaded by any person or persons to dim the lustre of the character of the corteous and gentlemanly chairman of the Executive Committee. The facts of the case are these: I was invited to attend the sessions at the Ocean Hotel, being a boarder at the hotel at that time. The best of feeling seemed to prevail among all the profession in the morning of the first day. As I went into the room where the convention met in congress, I noticed that the room was draped in deep mourning. I asked for whom, and was informed for their late Vice-President of the convention. Everything went lovely, until some member arose, and from what I now can recall to memory, I think he called for the drawing up of a set of resolutions in respect to their dead fellow and officer—

his motion was seconded, etc., but it was not entertained,—I saw some angry looks, but paid no attention, and not until recess did I become aware of the true facts of the case As the members of the Academy went from the session room, they were greeted with the "Extra News!" full account of the convention! all the speeches! from a newsboy—and purchasing a copy, they found a full and verbatim report of the speech which was not over five minutes finished. Now this enterprise of a journal would not surprise one in the metropolis, but for this little sheet with its two compositors and hand press, astonished us New York Bohemians. It was now that the growls began, and it was now for the first time that the dental gentlemen and "The Wanderer," was thrown some light. Why—honor and respect was not accorded the memory of the dead Vice-President and those gentleman who offered the "memoir." It was plain to be seen that a "slate" had been made, and somebody was afraid it would be broken. It was calculated (this is what I heard by eaves-dropping on the balcony of the hotel,) by somebody that his speech would be finished by a certain time. So the *Local* was bought by an exchange. The speech handed early in the morning long before the convention convened, and every thing fixed that the paper contained that speech in full, would be on the bluff at the hotels a half an hour after the close of its delivery in session. Now should these resolutions of "Memoir,' to the deceased fellow be entertained, over one hour would have been taken up, and a speech never spoken would be dished out to us at five pence a piece; this would'nt do, and this is the secret of the publication of that article. I saw there was a clique, and I sharpened my quill and went for them, for I hate rings. I was interviewed by the much abused gentleman, and I informed him that I could not gratify his wish to publish a denial, but that I would publish his denial, or that I would pay no attention, should he deny the charge, providing he would not libel me. I have letters from him in which he offers me a recompense should I publish a denial of the facts; and in these letters this person—frames for me—informs me what to say, how to word it, (the denial) and the excuses to make for my not denying it ere this; and should any more controversy arise from this, I will feel it my bounden duty to tell all I know, and publish all letters I have from that person. It was not until I was in the remote wilds of the far west that this person attacked me in your journal, and he never thought I would see it. I did not return until lately.

Very truly yours,

"WILL J. KELLETT," "*Wandering Willie,*"

84 Carleton Ave., Brooklyn.

